# Genetic and Evolutionary Analysis of Porcine Kobuvirus in Guangxi Province, Southern China, Between 2021 and 2025

**DOI:** 10.3390/microorganisms13081921

**Published:** 2025-08-17

**Authors:** Yang Tang, Yuwen Shi, Kaichuang Shi, Yanwen Yin, Shuping Feng, Feng Long, Hongbin Si

**Affiliations:** 1 College of Animal Science and Technology, Guangxi University, Nanning 530005, China; 18020762192@163.com (Y.T.); shiyuwen2@126.com (Y.S.); 2 Guangxi Center for Animal Disease Control and Prevention, Nanning 530001, China; yanwen0349@126.com (Y.Y.); fsp166@163.com (S.F.); longfeng1136@163.com (F.L.)

**Keywords:** porcine kobuvirus, *VP1* gene, *2B* gene, *3D* gene, phylogenetic analysis, genetic evolution

## Abstract

Kobuvirus is a new genus of viruses in the *Picornaviridae* family causing diarrhea in animals. Porcine kobuvirus (PKV) is an important pathogen with a high rate of infection in pig herds. In this study, a total of 10,990 fecal swabs and tissue samples were collected from different areas of Guangxi province in southern China during 2021–2025 and then tested for PKV using RT-qPCR. The results showed a 19.19% (2109/10,990) PKV positivity rate. Sixty-two PKV-positive samples, which were selected according to sampling regions, sampling seasons, and detection Ct values, were used for PCR amplification and gene sequencing. A sequence comparison showed that the nucleotide and amino acid identities of *VP1*, *2B*, and *3D* genes were 78.6–99.5% and 83.5–100%, 77.7–99.8% and 80.9–100%, and 90.9–99.8% and 94.9–99.9%, respectively, indicating that the *3D* gene was more conserved than the *VP1* and *2B* genes. The phylogenetic trees based on these three genes revealed that the PKV *VP1* gene sequences from different countries could be classified into two groups (Groups I and II), and the PKV *VP1* gene sequences obtained from Guangxi province were distributed in Groups I and II and formed independent clades. The *2B* and *3D* gene sequences could also be classified into two groups (Groups I and II). Bayesian analysis indicated a state of population growth for PKV strains from the time of their discovery until 2009, at which point it began to decline. Amino acid sequence analysis of the *VP1* gene identified mutations and insertions in the obtained PKV strains. Recombinant analysis showed that no recombinant event was found in the *VP1*, *2B*, and *3D* genes of the obtained strains. The results indicated the geographically specific inheritance and variation in PKV, provided more information on the prevalence and genetic evolution of PKV in Guangxi province, Southern China, and emphasized the importance of regularly monitoring genetic variation in PKV for better comprehension of PKV.

## 1. Introduction

The outbreaks of porcine diarrhea induced by viral pathogens in China have caused serious economic losses to the pig industry in recent years. Among these pathogens, traditional viruses, such as porcine epidemic diarrhea virus (PEDV), porcine rotavirus (PoRV), porcine transmissible gastroenteritis virus (TGEV), and porcine deltacoronavirus (PDCoV), and emerging viruses such as porcine astrovirus (PoAstV), porcine sapovirus (PoSaV), porcine norovirus (PoNoV), porcine sapelovirus (PSV), porcine kobuvirus (PKV), porcine teschovirus (PTV), and porcine enterovirus G (EV-G), are important causative agents capable of causing diarrhea in pigs [1,2,3]. Of these pathogens, PKV is transmitted via the fecal–oral route (through contaminated food and/or water) and often causes diarrhea or subclinical signs in infected piglets [4,5,6]. PKV is a member of the *kobuvirus* genus in the *Picornaviridae* family (http://talk.ictvonline.org/taxonomy/, accessed on 1 August 2025) [7]. It is a single-stranded positive-sense RNA virus with a spherical, icosahedrally symmetrical genome of about 8.2–8.3 kb in size. The genome includes a non-coding region at the 5′ end, an open reading frame (ORF) encoding a polyprotein, and a non-coding region at the 3′ end containing a poly (A) tail. The polyprotein is further hydrolyzed into a lead protein L (leader), three structural proteins VP0, VP3, and VP1, and seven non-structural proteins 2A, 2B, 2C, 3A, 3B, 3C, and 3D [8]. PKV was first discovered in Hungary in 2007 [4] and has since been detected in healthy and diarrheic pigs in many countries around the world [5,9], such as Thailand [10], Denmark [11], Japan [12], South Korea [13], China [14], France [15], Vietnam [16], Kenya [17], Brazil [18], the Netherlands [18], the United States (US) [19], Serbia [20], Italy [21], Mexico [22], the Czech Republic [23], and India [24]. Furthermore, PKV usually co-infects with other diarrhea-causing viruses [25,26,27,28,29]. For example, the samples of diarrheic piglets from 10 pig farms in Mexico showed that 36.49% (27/74) of the samples exhibited co-infection with PKV and PEDV [22], and the 543 fecal samples of diarrheic piglets from 22 provinces in China during 2015–2018 showed that co-infection with PKV was found to occur in up to 81.22% (441/543) of PEDV-positive samples [26], suggesting that PKV may exacerbate the PEDV-induced diarrheal signs [29]. In addition, sequence analysis in Korea indicated that one PKV strain (KU958660) was closely related to the bovine kobuvirus strain (HQ234909) [30], and the phylogenetic analysis of gene sequences from humans and various mammalian hosts (including bats, rodents, pigs, cats, and civets) revealed multiple cross-species transmissions within and among mammalian species [31]. These findings suggested that interspecies transmission between different animals, and even human–animal transmission, might occur in nature, and PKV might have zoonotic potential, which further emphasizes the importance of PKV prevention and control.

PKV infection seems to have an age dependence and is more frequently detected in suckling piglets (<28 days) [12,13,19,27,32], with positive infection rates ranging from 16.7% to 99.0% [33,34]. Since 2009, there has been an increasing trend of PKV positivity in China, and various PKV strains are prevalent in pig farms around the world, regardless of geographic location [5,9]. The coexistence of multiple strains has led to recombination events in PKV and has contributed to the genetic diversity and evolution of PKV [9,35]. Although PKV has been detected in fecal samples from both healthy and diarrheic piglets, the higher detection rate in diarrheic piglets compared to healthy piglets raises questions about the causative role of PKV [5,10,36,37,38]. The data showed that the PKV positivity rate was significantly higher in pigs with diarrheal sign than in subclinical pigs, which implies that it has pathogenicity for pigs [16,19,39,40]. However, the reports on the pathogenesis of PKV are still limited [41]. It is therefore necessary to study the prevalence and pathogenesis of PKV leading to swine diarrhea.

Guangxi province produces about 60 million pigs each year, so porcine diarrhea prevention and control are particularly important. PKV, as an emerging pathogen of porcine diarrhea, showed high infection rates in pig herds [32,33]. Currently, PKV cannot be successfully isolated and cultured, and conducting in-depth studies on its replication, assembly, and pathogenesis is not yet possible, so the exploration of PKV remains limited to epidemiological investigations and genome sequence analyses [20,39,42]. The PKV VP1 structural domain is the predominant immunogenic protein and the most variable region of viral genes, making it suitable for classifying PKV viruses [43,44]. The function of the 2B protein varies among different species of viruses in the *picornaviridae* family, but the specific function of the PKV 2B protein is still unclear. However, some scientists have classified PKV based on the presence of a 90-nucleotide (nt) deletion in the *2B* gene [40,45,46]. The 3D protein is an RNA-dependent RNA polymerase (RdRp) containing the highly conserved amino acid modules FLKR, YGDD, and KDELR, whose role is to participate in viral RNA replication [47]. Since the VP1, 2B, and 3D proteins of PKV play important and vital roles, they are usually taken as the target genes for analyzing genetic characteristics. According to previous reports, PKV had high positivity rates ranging from 1.46% (22/1502) to 21.72% (396/1823) in fecal samples from Guangxi province during 2023–2024 [48,49], so further epidemiological investigation of PKV was performed in this study. A total of 10,990 fecal swabs and intestinal tissue samples from 14 cities in Guangxi province during 2021–2025 were tested using RT-qPCR, and the PKV-positive samples were selected for *VP1*, *2B*, and *3D* gene sequence analysis to obtain detailed data on the molecular epidemiology of PKV in Guangxi province, southern China.

## 2. Materials and Methods

### 2.1. Collection and Detection of Clinical Samples for PKV

From March 2021 to February 2025, a total of 10,990 clinical samples, including 872 intestinal tissues and contents and 10,118 fecal swabs, were collected from pig farms, abattoirs, and harmless treatment plants in 14 prefecture-level cities in Guangxi province, southern China. According to the different clinical samples, the treatments were as follows: (1) Fecal swabs: 1.0 mL of PBS solution (pH 7.2) was put into a 2.0 mL EP tube containing fecal swabs, vortexed for 30 s, frozen-thawed three times, and then centrifuged (12,000 rpm for 5 min) at 4 °C to obtain the supernatant. (2) Intestinal tissues: the intestinal tissues and contents were put into a 2.0 mL EP tube; 1.0 mL of PBS solution and a sterilized steel ball were added; the solution was ground in an oscillating grinder (Retsch, MM400; Haan, Germany) for 5 min, frozen-thawed three times; and the solution was then centrifuged at 4 °C (12,000 rpm for 5 min) to obtain the supernatant. Nucleic acid extraction was performed using 200 µL of supernatants from tissue samples or fecal swabs using the MiniBEST Viral DNA/RNA Nucleic Acid Extraction Kit Ver.5.0 (TaKaRa, Dalian, China). Total DNA/RNA from clinical samples was used for detection of PKV using a quadruplex RT-qPCR, which was developed in our laboratory for the detection of PSV, PKV, PTV, and EV-G [49]. In brief, the forward primer PKV-F: CGTGCTGAGTAATGGGATAGG, the reward primer PKV-R: TGCACTTCAGAGGTCAGAGAA, and the TaqMan probe PKV-P: VIC-ATGAGTAGAGCATGGACTGCGGTG-BHQ1 were used to detect the 5′ UTR region of PKV with a 20 µL reaction system under 56 °C annealing and amplification temperature, and the samples with Ct values ≤ 36 were judged as positive samples. A total of 62 PKV-positive samples—based on their detection Ct values which were ≤30 (the average Ct value of these samples was 25.57), sampling locations from different cities in Guangxi province, and sampling time in different seasons of year—were then selected for *VP1*, *2B*, and *3D* gene amplification and sequence analysis.

### 2.2. Amplification and Sequencing

The specific primers were designed based on the PKV genome sequence (swine/S-1-HUN/2007/Hungary strain, GenBank accession number: EU787450) downloaded from the National Center for Biotechnology Information (NCBI, https://www.ncbi.nlm.nih.gov/, accessed on 12 September 2022) (Table 1). Total nucleic acids were extracted from the PKV-positive samples using the MiniBEST Viral DNA/RNA Nucleic Acid Extraction Kit Ver.5.0 (TaKaRa, Dalian, China), reverse-transcribed into cDNA using the PrimeScript™ II 1st Strand cDNA Synthesis Kit (TaKaRa, Dalian, China). The cDNAs were used as templates to amplify PKV *VP1*, *2B*, and *3D* gene fragments using the designed primers (Table 1). The PCR amplification system program was as follows: 25 μL of Premix Taq (Ex Taq Version 2.0 plus dye) (TaKaRa, Dalian, China), 0.8 μL each of forward and reward primers (20 μM), 5 μL of cDNA, and 18.4 μL of nuclease-free distilled water. The amplification procedures were as follows: *VP1*, *3D1*, and *3D2* gene fragments: 35 cycles of 94 °C for 3 min, 94 °C for 30 s, 58 °C for 30 s, and 72 °C for 70 s, followed by 10 min at 72 °C; *2B* gene fragment: 35 cycles of 94 °C for 3 min, 94 °C for 30 s, 64 °C for 30 s, 72 °C for 40 s, followed by 10 min at 72 °C.

The PCR products were purified using the MiniBEST DNA Fragment Purification Kit Ver.4.0 (TaKaRa, Dalian, China), ligated into the pMD18-T vector (TaKaRa, Dalian, China), and then transformed into *Escherichia coli.* DH5α competent cells (TaKaRa, Dalian, China). The positive clones were inoculated in LB medium containing ampicillin, cultured at 37 °C for 20–24 h, and sent to IGE Biotech (Guangzhou, China) for sequencing. Three clones were sequenced for each sample. After sequencing, the complete gene sequences of *VP1* and *2B* genes were obtained. The *3D1* and *3D2* gene sequences were spliced using the EditSeq tool in Lasergene DNAstar 7.0 (https://www.dnastar.com/software/, accessed on 12 September 2022) to obtain the complete gene sequences of the *3D* gene. Finally, the obtained gene sequences were compared and confirmed using the BLAST tool (https://blast.ncbi.nlm.nih.gov/Blast.cgi, accessed on 15 March 2025) on the NCBI website (https://www.ncbi.nlm.nih.gov/, accessed on 15 March 2025).

### 2.3. Sequence Comparison and Phylogenetic Analysis

The 102 *VP1*, 103 *2B*, and 103 *3D* gene sequences of PKV from Guangxi province and other provinces in China, as well as from Spain, Hungary, US, South Africa, Germany, and Japan, published in NCBI (https://www.ncbi.nlm.nih.gov/, accessed on 12 September 2022) were downloaded as reference sequences. The 62 *VP1*, 62 *2B*, and 62 *3D* gene sequences obtained in this study, together with the 102 *VP1*, 103 *2B*, and 103 *3D* reference sequences, i.e., 164 *VP1*, 165 *2B*, and 165 *3D* gene sequences (Supplementary Tables S1 and S2), were analyzed. The nucleotide and amino acid identities between the obtained sequences and the reference sequences were analyzed using the Clustal W algorithm in Bioedit v7.2.5 software (https://www.bioedit.com/, accessed on 12 September 2022). Phylogenetic trees of *VP1*, *2B*, and *3D* gene nucleotide sequences were constructed based on the maximum likelihood (ML) model in MEGA.X v10.26 software (https://www.megasoftware.net/dload_win_gui, accessed on 12 September 2022). The robustness of the phylogenetic trees was assessed via bootstrapping with 1000 replicates, and the phylogenetic tree optimization was performed using the online Interactive Tree of Life (iTOL) tool (https://itol.embl.de/, accessed on 15 March 2025). The genetic evolution rates of the *VP1*, *2B*, and *3D* genes were analyzed using the BEAST v1.10.4 software program (http://beast.community/, accessed on 12 September 2022).

### 2.4. Bayesian Dynamic Analysis of the VP1 Gene

The *VP1* gene encodes the predominant immunogenic protein and is the most variable region of viral genes [43,44], so it is selected for Bayesian dynamic analysis. The 62 PKV *VP1* sequences obtained in this study (Supplementary Table S2) and the 40 reference sequences (Supplementary Table S3) downloaded from NCBI were aligned using the MEGA.X v10.26 software program (https://www.megasoftware.net/dload_win_gui, accessed on 12 September 2022). Suitable substitution models were calculated via the ModelFinder tool of the PhyloSuite v1.2.2 software package (http://phylosuite.jushengwu.com, accessed on 12 September 2022). For the *VP1* gene, the relaxed molecular clock, GTR+F+G4 substitution model, and Bayesian SkyGrid model were finally selected, followed by 200 million steps of Markov chain Monte Carlo (MCMC) in parallel on three strands with a burn-in of 10%. After the run, the data was visualized with the Tracer v1.6 software program (https://beast.community/figtree, accessed on 12 September 2022) to obtain the gene evolution rate, and the convergence of all parameters was visually confirmed, with ESS > 200 considered valid for the parameters. The data were computed using Tree Annotator v1.10.4 (https://beast.community/treeannotator, accessed on 12 September 2022) to obtain the maximum clade confidence (MCC) tree, which was annotated after 10% aging and visualized using FigTree version 1.4.4 (https://beast.community/figtree, accessed on 12 September 2022).

### 2.5. Recombinant Analysis of the VP1, 2B, and 3D Genes

The 164 *VP1*, 165 *2B*, and 165 *3D* gene sequences of the 62 PKV strains obtained in this study and the reference strains downloaded from NCBI (https://www.ncbi.nlm.nih.gov/, accessed on 12 September 2022) (Supplementary Tables S1 and S2) were compared using the BioEdit v7.2.5 software program (https://www.bioedit.com/, accessed on 12 September 2022). All sequences were analyzed for recombination event using Recombination Detection Program (RDP4) (https://health.uct.ac.za/computational-biology, accessed on 12 September 2022). Seven methods were used to detect recombination events and breakpoints: RDP, BootScan, Chimaera, MaxChi, SiScan, GENECONV, and 3Seq. The sequences were analyzed for recombination event using the default settings of these seven algorithms, according to the recommendations of the RDP manual. The sequences supported by at least six algorithms were considered potentially recombinant sequences. Putative recombination events were further verified using the SimPlot v3.5.1 software program (https://github.com/Stephane-S/Simplot_PlusPlus, accessed on 12 September 2022).

## 3. Results

### 3.1. Detection of Clinical Samples

**Figure 1:**
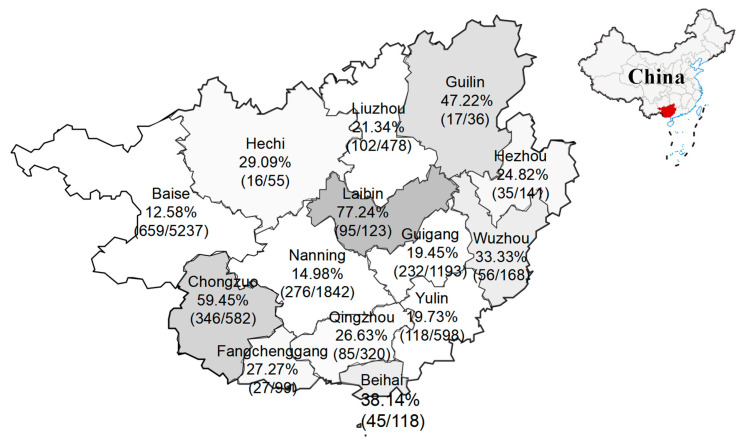
The distribution of PKV-positive samples in Guangxi province, southern China. The clinical samples were collected during 2021–2025, and the PKV positivity rates are marked for different regions.

The 10,990 clinical samples collected from Guangxi province during 2021–2025 were tested using the RT-qPCR developed in our laboratory [49]. The results showed that 2109 samples (19.19%, 2109/10,990) were positive for PKV, including 1944 fecal samples (19.21%, 1944/10,118) and 165 tissue samples (18.92%, 165/872). From 2021 to 2025, the PKV positivity rate in the clinical samples was 26.47% (221/835), 14.87% (609/4095), 18.99% (570/3001), 24.25% (651/2685), and 39.84% (149/374), respectively. PKV was detected in all 14 prefecture-level cities in Guangxi province with positivity rates ranging from 12.58% to 77.24%. The distribution of positive samples in different regions of Guangxi province is shown in Figure 1. A total of 62 positive samples were selected for *VP1*, *2B*, and *3D* gene amplification and sequencing. Finally, 62 *VP1*, 62 *2B*, and 62 *3D* gene sequences were obtained, and uploaded to GenBank in NCBI under the accession number of PV369260-PV369321 for the *VP1* gene, PV369322-PV369383 for the *2B* gene, and PV369384-PV369445 for the *3D* gene (Supplementary Table S2).

### 3.2. Identity Analysis of VP1, 2B, and 3D Genes

The nucleotide and amino acid sequences of the *VP1*, *2B*, and *3D* genes obtained in this study, as well as those of the reference strains, were analyzed using the Clustal W algorithm in BioEdit (https://www.bioedit.com/, accessed on 12 September 2022). The results showed that the identities of the nucleotide and amino acid sequences of the *VP1*, *2B*, and *3D* genes between the 62 PKV strains obtained in this study were 78.6–99.5% and 83.5–100%, 77.7–99.8% and 80.9–100%, and 90.9–99.8% and 94.9–99.9%, respectively. In addition, the identities of the nucleotide and amino acid sequences of the *VP1*, *2B*, and *3D* genes between the obtained 62 strains and the reference strains were 77.4–91.4% and 82.3–98.0%, 69.4–95.4% and 71.6–100%, and 89.7–95.0% and 94.4–99.8%, respectively.

### 3.3. Phylogenetic Analysis Based on VP1 Gene Sequences

**Figure 2:**
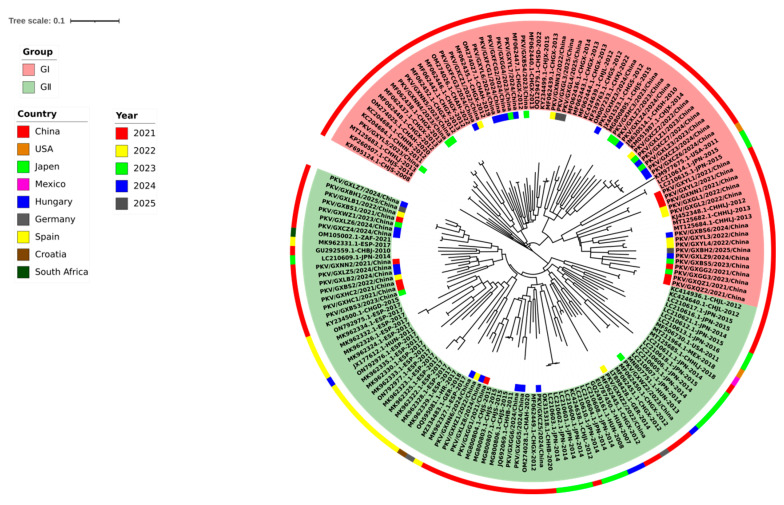
Phylogenetic tree based on the nucleotide sequences of the PKV *VP1* gene. In the inner ring, the PKV *VP1* gene sequences obtained in this study are marked with different-colored squares for different years. In the outer ring, the PKV strains from different countries are marked with different colors. CH: China; JPN: Japan; HUN: Hungary; ESP: The Kingdom of Spain; USA: The United States of America; GER: Germany; HR: The Republic of Croatia; MEX: The United Mexican States; ZAF: The Republic of South Africa.

To understand the phylogenetic information of the 62 *VP1* gene sequences obtained in this study, together with the 102 reference *VP1* gene sequences from Guangxi province, other provinces in China, and other countries, we constructed a phylogenetic tree using the GTR+G+I model, maximum likelihood (ML), and 1000 bootstrap tests (Figure 2). Since there is no standard method for systematic grouping of PKV, the groups of the phylogenetic trees were classified by referring to previous reports [46,47]. All 164 *VP1* gene sequences could be classified into two groups, i.e., Group I and Group II, and each group could be further divided into different clades. The strains in Group I originated from Asia and America, while the strains in Group II originated from Asia, Europe, and America. Most of the strains (41 strains) obtained in this study were distributed in Group I, while the other strains (21 strains) were distributed in Group II, and some of them formed independent clades (Figure 2).

### 3.4. Phylogenetic Analysis Based on 2B Gene Sequences

**Figure 3:**
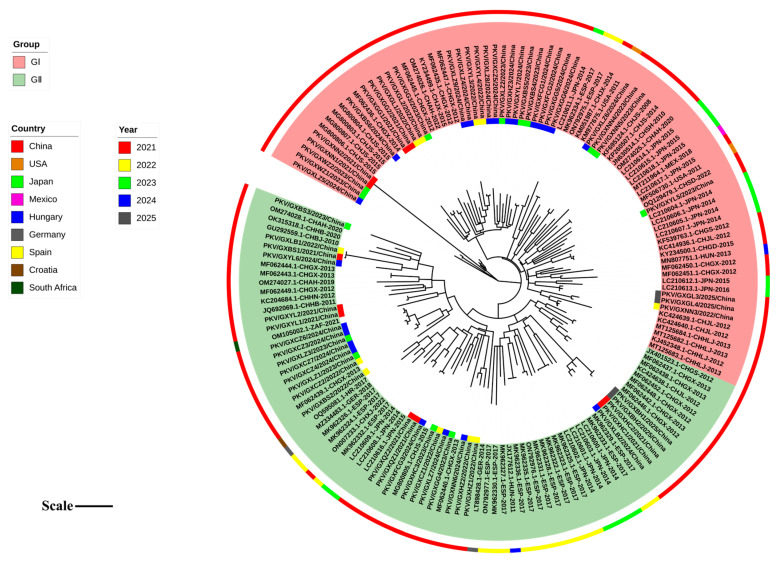
Phylogenetic tree based on the nucleotide sequences of the PKV *2B* gene. In the inner ring, the PKV *2B* gene sequences obtained in this study are marked with different-colored squares for different years. In the outer ring, the PKV strains from different countries are marked with different colors. Tree scale: 0.1. CH: China; JPN: Japan; HUN: Hungary; ESP: The Kingdom of Spain; USA: The United States of America; GER: Germany; HR: The Republic of Croatia; MEX: The United Mexican States; ZAF: The Republic of South Africa.

Based on the optimal nucleotide substitution model, GTR+G+I, a phylogenetic tree of the *2B* gene was generated using the maximum likelihood (ML) method with 1000 bootstrap tests (Figure 3). The phylogenetic tree was constructed based on the 62 sequences obtained in this study, and 103 reference sequences from Guangxi province, other provinces in China, and other countries. All *2B* gene sequences could be divided into two groups: Group I strains included those from Asia, including China and Japan; Group II strains included those from Asia, Europe, America, Africa, including China, Spain, Hungary, Germany, USA, Mexico, and South Africa. Each group could be further divided into different clades. The strains obtained in this study were distributed in Groups I and II, and some of them formed independent clades (Figure 3).

### 3.5. Phylogenetic Analysis Based on 3D Gene Sequences

**Figure 4:**
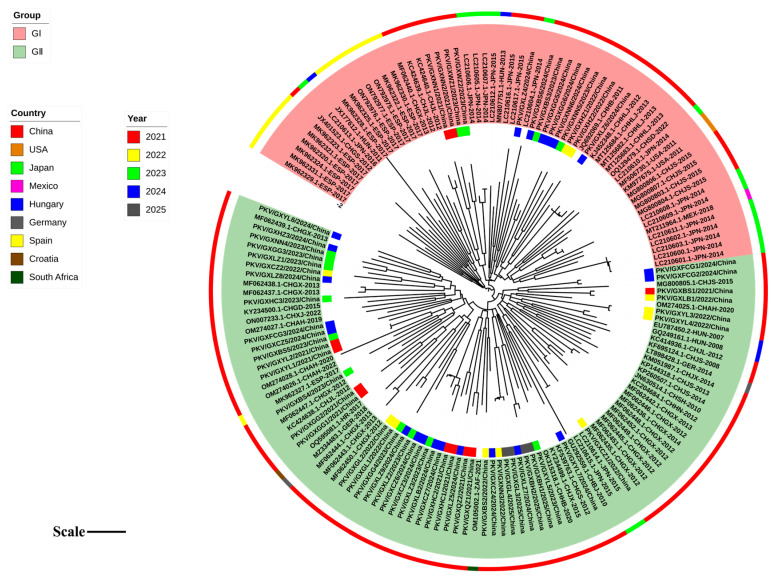
Phylogenetic tree based on the nucleotide sequences of the PKV *3D* gene. In the inner ring, the PKV *3D* gene sequences obtained in this study are marked with different-colored squares for different years. In the outer ring, the PKV strains from different countries are marked with different colors. Tree scale: 0.1. CH: China; JPN: Japan; HUN: Hungary; ESP: The Kingdom of Spain; USA: The United States of America; GER: Germany; HR: The Republic of Croatia; MEX: The United Mexican States; ZAF: The Republic of South Africa.

Based on the optimal nucleotide substitution model, GTR+G+I, a phylogenetic tree was constructed from the *3D* gene sequences of 62 PKV strains obtained in this study and 103 reference PKV strains using the maximum likelihood (ML) method with 1000 bootstrap tests (Figure 4). The phylogenetic tree revealed that all the sequences could be divided into two groups, Group I and Group II, and each group could be further divided into different clades. Group I included the strains from Asia, including China and Japan; Group II included the strains from Asia, Europe, and America, including China, Japan, Spain, Hungary, Germany, USA, and Mexico. The strains obtained in this study were distributed in Groups I and II. Some strains from Guangxi province, together with other Chinese strains, formed independent clades (Figure 4).

### 3.6. Bayesian Temporal Dynamic Analysis

**Figure 5:**
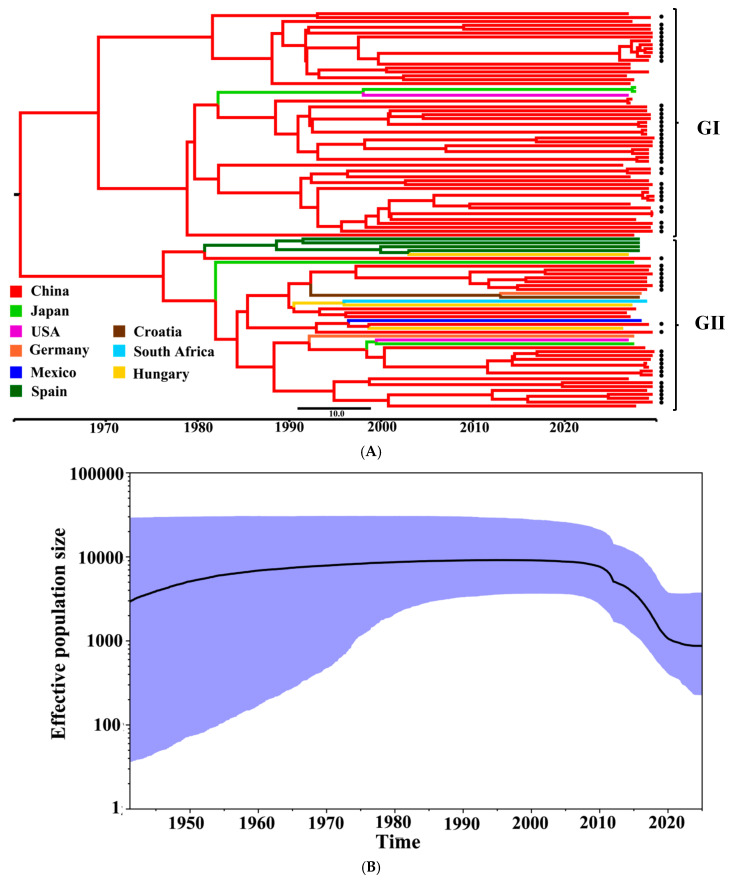
(**A**) The Maximum Clade Credibility (MCC) tree based on the nucleotide sequence of the PKV *VP1* gene, with clades in different colors representing different countries. The *VP1* gene sequences obtained in this study are marked with black dots. (**B**) Bayesian Skygrid of the PKV *VP1* gene. The dark blue line indicates mean values of genetic diversity, and the light blue shading indicates 95% confidence intervals.

The phylogenetic tree constructed based on the PKV *VP1* gene and the PKV time scale was consistent with the MCC tree (Figure 5). The MCC tree indicated that all the 102 obtained and reference PKV strains were classified into two groups (Groups I and II), and each group could be further divided into different clades (Figure 5A). The obtained 62 PKV strains were distributed in both Group I (41 strains) and Group II (21 strains), and some strains formed independent clades. The generated Bayesian Skygrid plot visualizes the actual population size and spread of PKV in Guangxi province in recent years, revealing the time-historical origin of PKV based on the *VP1* gene (Figure 5B). The Bayesian Skygrid plot revealed that the effective population size of PKV had been increasing since 1990s, followed by a decline starting around 2009 and continuing to the present day.

### 3.7. Recombination Analysis of the VP1, 2B, and 3D Genes

Recombination event analysis of the 164 *VP1*, 165 *2B*, and 165 *3D* gene sequences of the obtained strains and the reference strains was performed using the RDP4 software program, and no recombination event was found in these gene sequences.

### 3.8. Estimation of Evolution Rates of VP1, 2B, and 3D Genes

The genetic evolutionary rates of PKV 164 *VP1*, 165 *2B*, and 165 *3D* genes were analyzed using BEAST software. The results showed that the evolutionary rates of *VP1*, *2B*, and *3D* genes were 3.72 × 10^−3^, 2.50 × 10^−2^, and 2.88 × 10^−4^ substitution/site/year (s/s/y), respectively, indicating that the *2B* gene had the highest evolutionary rate (Table 2).

### 3.9. Analysis of VP1 Gene Amino Acid Sequences

To further explore the genetic characteristics of PKV strains, the obtained 62 VP1 amino acid sequences distributed in two groups of the phylogenetic tree were entered into BioEdit (https://www.bioedit.com/, accessed on 12 September 2022) for comparison and analysis. The VP1 amino acid sequences of 41 strains in Group I obtained in this study were compared with the amino acid sequences of the reference strain (SH-W-CHN/2010/China strain, GenBank accession number: JN630514), and the amino acid sequences of 21 strains in Group II obtained in this study were compared with the amino acid sequences of the reference strain (Y-1-CHI strain, GenBank accession number: GU292559). The results showed that the obtained sequences of Group I were found to contain 88 mutations and one insertion, including 21 high-frequency conserved mutation sites; the obtained sequences of Group II contained 45 mutations and one insertion, with 16 identical mutation sites. These identical mutation sites are presented in Supplementary Figure S1, and all mutation sites are listed in detail in Table 3.

## 4. Discussion

Newly emerging viral pathogens have played important roles in inducing diarrhea in piglets in recent years [2,3]. PKV has been identified as one of the enteric viruses prevalent worldwide [4,5,6,32,33], and the co-infection of PKV with other enteric viruses can exacerbate diarrhea diseases [25,26,27,28,29,32]. PKV has therefore attracted widespread attention. In this study, 10,990 clinical samples collected from Guangxi province during 2021–2025 were tested for PKV using RT-qPCR. The PKV positivity rate was 19.19% (2109/10,990), ranging from 14.87% to 39.84% during 2021–2025, thus confirming the high prevalence rate of PKV in Guangxi province. In China, PKV has also been reported in other provinces with different positivity rates, ranging from 29.94% (94/314) to 81.22% (441/543) [14,39,40,46,50,51]. Besides China, PKV has also been reported in many other countries [1,5,9,10,11,12,13,15,16,17,18,19,20,21,22,23,24], with a positivity rate of 45.39% (133/293) in Japan [12], 36.13% in Korea [13], 44.82% (407/908) in France [15], 29.33% (200/682) in Vietnam [16], 13.15% (33/251) in East Africa [17], 53.04% (61/115) in Brazil [18], 22% (22/100) in Serbia [20], 51.35% (38/74) in Mexico [22], 87.24% (171/196) in the Czech Republic [23], 95.75% (609/636) in Northern Thailand [30], and so on. As it is widespread, PKV should be paid more research attention, even if it is usually associated with subclinical signs in infected pigs, and the pathogenicity and pathogenesis still need to be further researched and determined.

A total of 62 PKV-positive samples were selected for the amplification and sequencing of *VP1*, *2B*, and *3D* genes, and the obtained sequences were compared with the representative reference strains from different countries. The nucleotide and amino acid identities of *VP1*, *2B*, and *3D* genes among the obtained 62 PKV strains were 78.6–99.5% and 83.5–100%, 77.7–99.8% and 80.9–100%, 90.9–99.8% and 94.9–99.9%, indicating that the *3D* gene had the highest identity in these three genes. The nucleotide and amino acid identities of *VP1*, *2B*, and *3D* genes between the obtained 62 PKV strains and the reference strains were 77.4–91.4% and 82.3–98.0%, 69.4–95.4% and 71.6–100%, and 89.7–95.0% and 94.4–99.8%, respectively, indicating that the *3D* gene also showed the highest identity in these three genes. The homology of these genes among PKV strains from different countries has been reported previously, and the identity rates were similar to the results in this study [13,27,46,51,52,53], indicating that there existed genetic differences in PKV prevalent strains in different countries.

The phylogenetic trees based on 164 *VP1*, 165 *2B*, and 165 *3D* gene sequences could classify the PKV strains from different countries into two groups, respectively, which was similar to previous reports [9,12,16,22,27,32,33,39,40,46,52]. It is noteworthy that some PKV strains might be distributed in different group based on different gene sequences (Supplementary Tables S1 and S2). The obtained 62 strains in this study were distributed in different groups. The phylogenetic analysis based on *VP1* gene sequences revealed that all PKV strains could be divided into two groups, and the 62 strains obtained in this study were distributed in these two groups, each of which exhibited characteristic patterns of molecular variation. From the 41 obtained strains in Group I, 88 amino acid site mutations and one site insertion (Q234V/S/I/A/T/L) were identified, as well as existing 21 high-frequency conserved mutation sites (S12N, L24P, D54N, I105L, E131D/N/G, A146V/T, A154I/V/T, V160I, Q183H/R, S198N/T/A, A205S/I/T, M208L/Y, T213A, C221N, E232G, Q233A/T, P236T/S/V, L238P/S/A, L239A/V/T/I, P240A/V/S/T, and V251I) (Table 3). The C-terminus of VP1 is usually involved in the multimerization of coat proteins, and a large number of amino acid mutations at the C-terminus in Group I might affect the stability of PKV strains. The 21 obtained strains in Group II exhibited 45 site mutations and one site (237P) insertion, and the 16 characteristic mutations (P8T, Q51E, I80N/V, S86T, A99G, V117I, G130T/N, T146I/V/A, I151T, I158V, V197A, S198A/V/T, A205N/T/S, I206P/A, I236T/A, and T241A) (Table 3) are significantly different from those of Group I, reflecting different evolutionary characteristics of different groups. Systematic comparison of mutation patterns among multiple groups suggests that PKV strains circulating in Guangxi province might have acquired adaptive mutations through different evolutionary pathways, and the mutations might contribute to the high level of PKV genetic diversity. Previous reports showed that some circulating PKV strains had 90 nt deletion in *2B* gene [40,45,46], but none of the 62 obtained strains in this study showed this deletion. In addition, recombinant analysis of 164 *VP1*, 165 *2B*, and 165 *3D* gene sequences of the obtained strains and the reference strains was performed, but no recombination event was found, suggesting that recombination might not be the main mechanism to induce the variation in PKV, even if a recombinant event in PKV has been reported [9,40,54]. Mutations, deletion, and insertions might mainly contribute to the high level of genetic diversity in PKV [40,50,52].

Bayesian phylogenetic analysis was performed on the 164 *VP1*, 165 *2B*, and 165 *3D* gene sequences from different countries, and showed genetic evolution rates of 3.72 × 10^−3^, 2.50 × 10^−2^, and 2.88 × 10^−4^ s/s/y for three genes, respectively, indicating a high rate of variation in the PKV *VP1*, *2B*, and *3D* genes. The results showed that the *2B* gene had the highest evolution rate, and the *3D* gene had the lowest evolution rate. The evolutionary rates of *VP1* (3.72 × 10^−3^ s/s/y) and *3D* (2.88 × 10^−4^ s/s/y) genes estimated in this study were slightly higher than that of *VP1* gene (2.19 × 10^−3^ s/s/y) reported in Vietnam [55], but significantly lower than that of *VP1* gene (2.73 × 10^−2^ s/s/y), and the *3D* gene (1.30 × 10^−2^ s/s/y) reported in Korea [56,57]. The Bayesian skyline model analysis showed that the effective population size of PKV in Guangxi province has shown an increasing and then decreasing trend since 1990. This dynamic trend is closely related to the development of the local pig farming industry and the history of disease prevention and control. In the early 1990s, pig farming in China was still based on the free-range model, with poor biosecurity measures and a high rate of vaccine immunization failure against swine diseases, which led to epidemics of these diseases in Guangxi province. This situation created favorable conditions for the co-infection of PKV with other viruses, contributing to the continuous expansion of its effective population size. After 2009, this trend was significantly reversed, with the continuous improvement of the prevention and control system in the pig farming industry. The breakthrough of swine vaccine technology, the promotion of closed management mode in large-scale pig farms, the standardization of disinfection measures, and strict biosecurity measures have led to a greatly improved epidemic prevention and control system [58,59], effectively inhibiting the spread of PKV and ultimately reducing the effective population size of PKV.

The MCC tree mapping was performed on the 102 *VP1* gene sequences, and the typing results were consistent with the results of the phylogenetic trees. Group I consisted of most of the native Chinese strains, and such a high level of geographic characteristics suggested that this group might have evolved independently within China. Group II contained strains from China and other countries, but the strains obtained in this study showed strong genetic association with other Chinese strains, suggesting these obtained strains might have originated from the native strains in China. In addition, the results showed the Guangxi strains were closely related to the Hungarian strains, suggesting these strains might be originated from Europe. The results suggested that the PKV circulating in Guangxi province might be originated from other provinces of China and other countries, showing the complexity of their origins. Previous reports also suggested that the PKV strains circulating in other provinces in China might be originated from the native strains in China, and from other countries in America and Europe [39,40,46]. This situation might have contributed to the sources of breeding pigs in China, which were imported from Europe and America, with the pathogen being introduced into China via breeding pigs and spreading throughout the country.

## 5. Conclusions

The 10,990 clinical samples collected from Guangxi province during 2021–2025 showed a 19.19% positivity rate of PKV. Sixty-two positive samples were selected for amplification and sequence analysis of the PKV *VP1*, *2B*, and *3D* genes. The phylogenetic tree based on *VP1* gene sequences revealed that all PKV strains from different countries could be classified into two groups, and the 62 PKV strains obtained in this study were distributed in two groups. Amino acid sequence analysis showed many mutations located in different sites in the *VP1* gene. No recombination event was found in the PKV *VP1*, *2B*, and *3D* genes of the obtained 62 strains. Since its discovery in 2007, the effective population size of PKV in Guangxi province exhibited an increasing trend until around 2009, and a decline since then. These results are informative regarding the prevalence and genetic evolution of PKV in Guangxi province in southern China and contribute to a better understanding of PKV.

## Figures and Tables

**Table 1:** The primers for amplification of the PKV *VP1*, *2B*, and *3D* genes.

Gene	Primer	Sequence (5′→3′)	Product/bp
*VP1*	PKV-VP1-F	GTCACTAACATGGCTAACCAGAA	1162
	PKV-VP1-R	CCATCCAGTGACGTGGTTCTACCTC	
*2B*	PKV-2B-F	GCCGTGCAAGCGTCCAAAGG	654
	PKV-2B-R	CGCGTTGACAGCATCATTGTA	
*3D*	PKV-3D1-F	TCGAGCAGTTTGCGATTGACCAA	1048
	PKV-3D1-R	GGATGGATGGGCGGATCACACCC	
	PKV-3D2-F	GGTGGACTCATCGAGTACATGCA	899
	PKV-3D2-R	CGGTCTTAGGAAAGCATGAGTCTAT	

**Table 2:** The evolutionary rates of PKV *VP1*, *2B*, and *3D* genes.

Gene	Mean Evolutionary Rate (s/s/y)	95% Highest Posterior Density (HPD)
*VP1*	3.72 × 10^−3^	3.11 × 10^−3^–4.44 × 10^−3^
*2B*	2.50 × 10^−2^	1.42 × 10^−2^–3.93 × 10^−2^
*3D*	2.88 × 10^−4^	1.38 × 10^−4^–6.99 × 10^−4^

**Table 3:** Amino acid mutation profiles in *VP1* gene of the PKV strains obtained in this study.

Group	Site	Mutation	Site	Mutation
I	2	D→G	121	F→Y
	3	D→N/G	131	E→D/N/G
	4	D→A	132	E→Q
	5	N→D	134	P→S
	7	P→Q/T	135	G→S
	8	P→T	137	S→A/T
	12	S→N	139	G→S
	17	T→S	141	F→S
	18	A→T	144	I→V
	19	T→A	146	A→V/T
	20	T→S	148	I→V
	21	E→Q	151	T→A
	24	L→P	152	S→F
	28	F→L	154	A→I/V/T
	29	S→T	158	I→L
	44	F→S	160	V→I
	45	F→L	177	S→T
	52	F→Y	178	D→G
	54	D→N	181	G→S
	56	E→Q/D/G	183	Q→H/R
	57	D→N	188	T→A
	61	G→S	197	V→I/A
	65	E→G/A	198	S→N/T/A
	66	A→N/D/V	202	D→E/G
	69	T→A	205	A→S/I/T
	70	F→L	206	T→S/P/A
	71	P→Q/H	208	M→L/Y
	76	D→G/N	210	T→A
	80	N→T/I/V/M	213	T→A
	82	G→S	221	C→N
	86	T→A	231	L→I
	97	F→I	232	E→G
	99	A→V	233	Q→A/T
	104	A→T/V	234	V/I/S/A/N/L
	105	I→L	236	P→T/S/V
	107	F→L	237	P→A/V/S
	109	N→T	238	L→P/S/A
	112	A→P	239	L→A/V/T/I
	113	Y→F	240	P→A/V/S/T
	114	A→T	241	A→L/V
	115	A→V	250	P→R
	116	R→C/S	251	V→I
	117	V→I	252	V→T
	118	T→I	254	Q→R
	119	I→V		
II	5	D→N	137	S→P
	7	Q→E/P	146	T→I/V/A
	8	P→T	148	I→V/A
	17	S→T	151	I→T
	19	T→A	154	A→T/V
	38	D→G	156	I→V
	43	R→C	158	I→V
	56	Q→E/T	160	I→V
	66	A→T	173	F→L
	69	T→I	183	H→Q
	70	F→L/S	197	V→A
	71	P→H	198	S→A/V/T
	80	I→N/V	200	Q→L
	86	S→T	205	A→N/T/S
	96	Y→C	206	I→P/A
	99	A→G	210	T→I
	100	D→G	223	Y→F
	102	R→H	234	S→L/F/P
	113	Y→F	236	I→T/A
	115	A→V	237	/→P
	116	C→F/S	238	P→S
	117	V→I	239	A→V/T
	118	T→I	240	A→T
	130	G→T/N	241	T→A
	132	D→N	250	R→P
	135	T→I		

## Data Availability

The original contributions presented in this study are included in the article/Supplementary Materials. Further inquiries can be directed to the corresponding authors.

## Supplementary Materials

The following supporting information can be downloaded at https://www.mdpi.com/article/10.3390/microorganisms13081921/s1, Table S1: The information about the PKV reference strains used in this study. Table S2: The information about the PKV strains obtained in this study. Table S3. The information on the PKV reference strains used for Maximum Clade Credibility (MCC) tree. Figure S1: Amino acid mutation sites of the PKV *VP1* gene obtained in this study.
