# Supplementary material for: Genetic and Evolutionary Analysis of Porcine Kobuvirus in Guangxi Province, Southern China, Between 2021 and 2025
Source: Microorganisms. 2025 Aug 17;13(8):1921. doi: 10.3390/microorganisms13081921 (PMC12388615; doi:10.3390/microorganisms13081921)
Supplement: Supplementary file 1 [file microorganisms-13-01921-s001.zip › Supplementary Figure S1.pdf]

Supplementary Materials

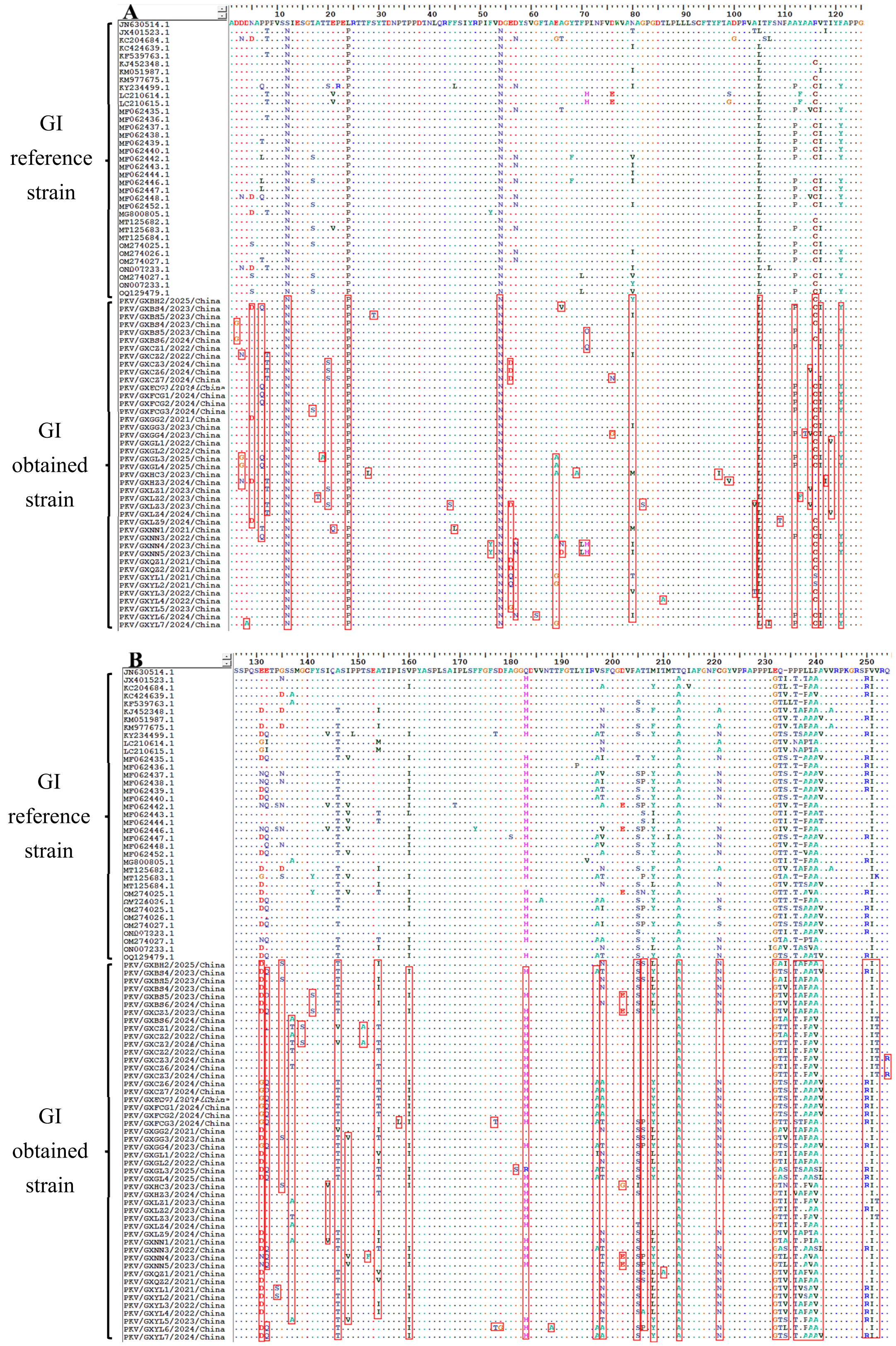

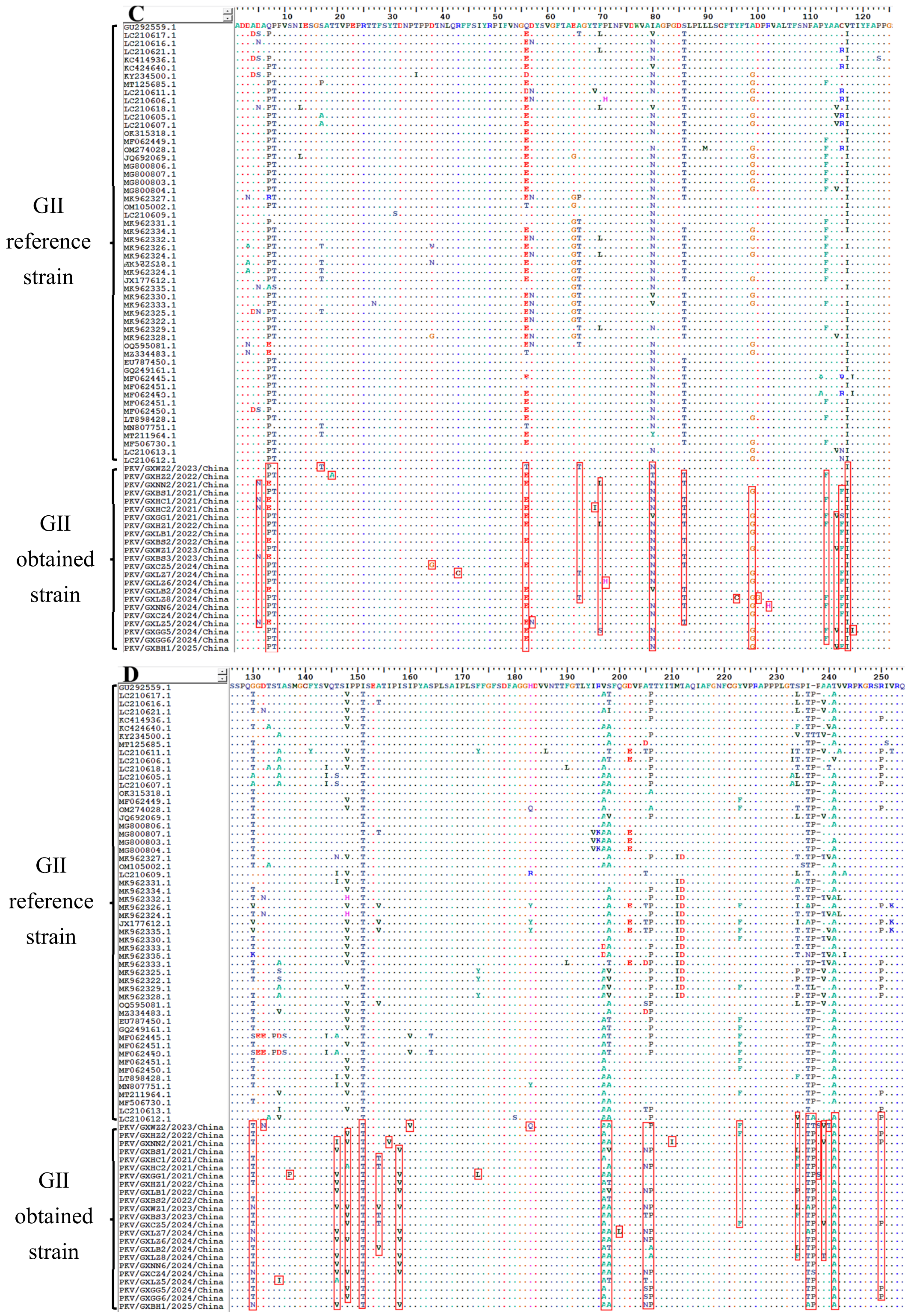
C
D
GII reference strain
GII obtained strain

Figure S1. Amino acid mutation sites of the PKV VP1 gene obtained in this study. The marked positions represent mutation sites presented in nearly all sequences. Panels A-B show Group I, and panels C-D show Group II.
