# Supplementary material for: Genetic and Evolutionary Analysis of Porcine Kobuvirus in Guangxi Province, Southern China, Between 2021 and 2025
Source: Microorganisms. 2025 Aug 17;13(8):1921. doi: 10.3390/microorganisms13081921 (PMC12388615; doi:10.3390/microorganisms13081921)
Supplement: Supplementary file 1 [file microorganisms-13-01921-s001.zip › Supplementary Table S1-S3.pdf]

Supplementary Materials

Table S1. The information on the PKV reference strains used in this study.

No.	Virus strain	Date	Origin	Accession Number	Group (VP1/2B/3D)
1	AH-42	2022	Anhui, CHN	OM274026	GI/GI/GII
2	AH-75	2020	Anhui, CHN	OM274028	GII/GII/GII
3	AH-49	2019	Anhui, CHN	OM274027	GI/GII/GII
4	AH-32	2020	Anhui, CHN	OM274025	GI/GI/GII
5	Y-1-CHI	2010	Beijing, CHN	GU292559	GII/GII/GII
6	CH/HNXX-4/2012	2012	Gansu, CHN	JX401523	GI/GII/GI
7	swKoV CH441	2012	Gansu, CHN	KF539763	GI/GI/GII
8	JXJC2015	2015	Guangdong, CHN	KY234500	GII/GII/GII
9	Guangxi/G6/2012/CH	2012	Guangxi, CHN	MF062450	GII/GII/GII
10	Guangxi/G2/2012/CH	2012	Guangxi, CHN	MF062452	GI/GII/GII
11	Guangxi/G9/2012/CH	2012	Guangxi, CHN	MF062447	GI/GI/GII
12	Guangxi/G21/2014/CH	2014	Guangxi, CHN	MF062436	GI/GII/GII
13	Guangxi/G14/2013/CH	2013	Guangxi, CHN	MF062443	GI/GII/GII
14	Guangxi/G5/2012/CH	2012	Guangxi, CHN	MF062451	GII/GII/GII
15	Guangxi/G7/2012/CH	2012	Guangxi, CHN	MF062449	GII/GII/GII
16	Guangxi/G19/2013/CH	2013	Guangxi, CHN	MF062438	GI/GII/GII
17	Guangxi/G8/2012/CH	2012	Guangxi, CHN	MF062448	GI/GII/GII
18	Guangxi/G20/2013/CH	2013	Guangxi, CHN	MF062437	GI/GII/GII
19	Guangxi/G11/2012/CH	2012	Guangxi, CHN	MF062446	GI/GII/GII
20	Guangxi/G15/2013/CH	2013	Guangxi, CHN	MF062442	GI/GII/GII
21	Guangxi/G17/2013/CH	2013	Guangxi, CHN	MF062440	GI/GII/GI
22	Guangxi/G1/2012/CH	2012	Guangxi, CHN	MF062435	GI/GI/GII
23	Guangxi/G13/2013/CH	2013	Guangxi, CHN	MF062444	GI/GII/GII
24	Guangxi/G18/2013/CH	2013	Guangxi, CHN	MF062439	GI/GII/GI
25	Guangxi/G12/2012/CH	2012	Guangxi, CHN	MF062445	GII/GI/GII
26	swine/HBYT/2018/China	2018	Heilongjiang, CHN	MT125685	GI/GII/GI
27	swine/JX-3/2013/China	2013	Heilongjiang, CHN	MT125684	GI/GI/GI
28	swine/JX-2/2013/China	2013	Heilongjiang, CHN	MT125683	GI/GI/GI
29	swine/JX-1/2013/China	2013	Heilongjiang, CHN	MT125682	GI/GI/GI
30	CH/DX/2012	2012	Heilongjiang, CHN	KJ452348	GI/GI/GI
31	GS-2/2012/CH	2012	Heilongjiang, CHN	KC424640	GII/GI/GI
32	GS-1/2012/CH	2012	Heilongjiang, CHN	KC424639	GI/GI/GI
33	K-4/2012/CH	2012	Heilongjiang, CHN	KC424638	GII/GI/GII
34	XX	2012	Henan, CHN	KC204684	GI/GII/GII
35	WUH1	2011	Hubei, CHN	JQ692069	GII/GII/GI
36	Wuhan2020	2020	Hubei, CHN	OK315318	GII/GII/GII
37	JS-01-CHN/2013/China	2013	Jiangsu, CHN	KP144318	×/×/GII
38	JS-02a-CHN/2014/China	2014	Jiangsu, CHN	KP260507	GI/GI/GII

39	ch-kobu/2008/China	2008	Jiangsu, CHN	KF695124	GI/GI/GII
40	Chgz5	2015	Jiangsu, CHN	MG800807	GII/GI/GI
41	Chgz4	2015	Jiangsu, CHN	MG800806	GII/GI/GI
42	Chgz3	2015	Jiangsu, CHN	MG800805	GI/GII/GII
43	Chgz2	2015	Jiangsu, CHN	MG800804	GII/GI/GI
44	Chgz1	2015	Jiangsu, CHN	MG800803	GII/GI/GI
45	CH/KB-1/2014	2014	Jiangxi, CHN	KM051987	GI/GI/GII
46	JXAT2015	2015	Jiangxi, CHN	KY234499	GI/GI/GII
47	K-11/2012/CH	2012	Jilin, CHN	KC414936	GII/GI/GII
48	SD/2022/China	2022	Shandong, CHN	OQ129479	GI/GI/GII
49	SH-W-CHN/2010/China	2010	Shanghai, CHN	JN630514	GI/GI/GII
50	BSF2	2021	ZAF	OM105002	GII/GII/GII
51	XJ1904-34-PKV	2022	Xinjiang, CHN	ON007233	GI/GII/GII
52	swine/S-1-HUN/2007/Hungary	2007	HUN	EU787450	GI/GII/GII
53	SP-VC36	2017	ESP	ON792977	GII/GII/GI
54	SP-VC18	2017	ESP	ON792976	GII/GII/GI
55	SP-VC4	2017	ESP	ON792975	GII/GI/GI
56	KobuV/Pig-wt/ESP/VC11/2017	2017	ESP	MK962336	GI/GII/GI
57	KobuV/Pig-wt/ESP/VC8/2017	2017	ESP	MK962335	GII/GII/GI
58	KobuV/Pig-wt/ESP/VC14/2017	2017	ESP	MK962334	GII/GI/GI
59	KobuV/Pig-wt/ESP/P461/2017	2017	ESP	MK962333	GII/GII/GI
60	KobuV/Pig-wt/ESP/C375/2017	2017	ESP	MK962332	GII/GII/GI
61	KobuV/Pig-wt/ESP/P284/2017	2017	ESP	MK962331	GII/GII/GI
62	KobuV/Pig-wt/ESP/P452/2017	2017	ESP	MK962330	GII/GII/GI
63	KobuV/Pig-wt/ESP/P2B/2017	2017	ESP	MK962329	GII/GII/GI
64	KobuV/Pig-wt/ESP/B304/2017	2017	ESP	MK962328	GII/GII/GI
65	KobuV/Pig-wt/ESP/P259/2017	2017	ESP	MK962327	GI/GII/GII
66	KobuV/Pig-wt/ESP/C429/2017	2017	ESP	MK962326	GII/GII/GI
67	KobuV/Pig-wt/ESP/VT25C/2017	2017	ESP	MK962325	GII/GII/GI
68	KobuV/Pig-wt/ESP/P437/2017	2017	ESP	MK962324	GII/GII/GI
69	KobuV/Pig-wt/ESP/P37/2017	2017	ESP	MK962323	GII/GII/GI
70	KobuV/Pig-wt/ESP/VC20B/2017	2017	ESP	MK962322	GII/GII/GI
71	KobuV/Pig-wt/ESP/P393/2017	2017	ESP	MK962321	GI/GII/GI
72	KobuV/Pig-wt/ESP/C247/2017	2017	ESP	MK962320	×/GII/GI
73	FoPro-213.14/GER/2018	2018	GER	MZ334483	GII/GII/GII
74	PKV_GER_L00919-K17_14-02_2014	2014	GER	LT898428	GII/GII/GII
75	06-2017-Medj	2017	HR	OQ595081	GII/GII/GII
76	K-30-HUN/2008/HUN	2008	HUN	GQ249161	GII/GII/×
77	SZ1M-F/PKV/HUN/2013	2013	HUN	MN807751	GII/GII/GI
78	WB-1-HUN/2011/HUN	2011	HUN	JX177612	GII/GII/GI
79	PoKoV/Bu4-4/JPN/2014	2014	JPN	LC210606	GII/GII/GI
80	PoKoV/Bu4-2/JPN/2014	2014	JPN	LC210605	GII/GII/GI
81	PoKoV/Bu3-5/JPN/2014	2014	JPN	LC210604	GII/GII/GI
82	PoKoV/Bu2-6/JPN/2014	2014	JPN	LC210603	GII/GII/GI
83	PoKoV/Bu2-5/JPN/2014	2014	JPN	LC210602	GII/GII/GI

84	PoKoV/Bu2-3/JPN/2014	2014	JPN	LC210601	GII/GII/GI
85	PoKoV/Bu2-2/JPN/2014	2014	JPN	LC210600	GII/GII/GI
86	PoKoV/Bu1-8/JPN/2014	2014	JPN	LC210599	GI/GII/GI
87	PoKoV/Tochigi-IO3/JPN/2014	2014	JPN	LC210621	GII/GII/GI
88	PoKoV/Tochigi-IN30/JPN/2015	2015	JPN	LC210620	GI/GII/GI
89	PoKoV/Ishi-Ta4/JPN/2015	2015	JPN	LC210619	GI/GII/GI
90	PoKoV/Ishi-Ta3/JPN/2015	2015	JPN	LC210618	GII/GI/GI
91	PoKoV/Ishi-Sa5/JPN/2015	2015	JPN	LC210617	GII/GI/GI
92	PoKoV/Ishi-Ka3/JPN/2015	2015	JPN	LC210616	GII/GII/GI
93	PoKoV/Ishi-Im6/JPN/2015	2015	JPN	LC210615	GI/GI/GII
94	PoKoV/Ishi-Im5/JPN/2015	2015	JPN	LC210614	GI/GI/GII
95	PoKoV/Iba444-2/JPN/2016	2016	JPN	LC210613	GII/GI/GI
96	PoKoV/Iba27-372/JPN/2015	2015	JPN	LC210612	GII/GI/GI
97	PoKoV/Bu9-10/JPN/2014	2014	JPN	LC210611	GII/GII/GI
98	PoKoV/Bu7-9/JPN/2014	2014	JPN	LC210610	GII/GII/GI
99	PoKoV/Bu6-6/JPN/2014	2014	JPN	LC210609	GII/GII/GI
100	PoKoV/Bu5-6/JPN/2014	2014	JPN	LC210608	GII/GII/GI
101	PoKoV/Bu4-6/JPN/2014	2014	JPN	LC210607	GII/GII/GI
102	EdoMex/2018/205	2018	MEX	MT211964	GI/GI/GI
103	OH/RV50/2011	2011	USA	KM977675	GI/GI/GI
104	OH/RV11/2011	2011	USA	MF506730	GII/GI/GI

Note: CHN: China; ESP: The Kingdom of Spain; GER: Germany; HR: The Republic of Croatia; HUN: Hungary; JPN: Japan; MEX: The United Mexican States; USA: The United States of America; ZAF: The Republic of South Africa. The same as below.

Table S2. The information on the PKV strains obtained in this study.

No.	Virus strain	Date	Origin	Accession Number			Group
				VP1	2B	3D	(VP1/2B/3D)
1	PKV/CHGXNN1/2021	2021	Guangxi, CHN	PV369260	PV369322	PV369384	GI/GI/GI
2	PKV/CHGXNN2/2021	2021	Guangxi, CHN	PV369261	PV369323	PV369385	GII/GI/GI
3	PKV/CHGXNN3/2022	2022	Guangxi, CHN	PV369262	PV369324	PV369386	GI/GI/GII
4	PKV/CHGXNN4/2023	2023	Guangxi, CHN	PV369263	PV369325	PV369387	GI/GI/GII
5	PKV/CHGXNN5/2023	2023	Guangxi, CHN	PV369264	PV369326	PV369388	GI/GI/GI
6	PKV/CHGXNN6/2024	2024	Guangxi, CHN	PV369265	PV369327	PV369389	GII/GII/GI
7	PKV/CHGXYL1/2021	2021	Guangxi, CHN	PV369266	PV369328	PV369390	GI/GII/GII
8	PKV/CHGXYL2/2021	2021	Guangxi, CHN	PV369267	PV369329	PV369391	GI/GII/GII
9	PKV/CHGXYL3/2022	2022	Guangxi, CHN	PV369268	PV369330	PV369392	GI/GII/GII
10	PKV/CHGXYL4/2022	2022	Guangxi, CHN	PV369269	PV369331	PV369393	GI/GI/GII
11	PKV/CHGXYL5/2023	2023	Guangxi, CHN	PV369270	PV369332	PV369394	GI/GI/GII
12	PKV/CHGXYL6/2024	2024	Guangxi, CHN	PV369271	PV369333	PV369395	GI/GII/GII
13	PKV/CHGXYL7/2024	2024	Guangxi, CHN	PV369272	PV369334	PV369396	GI/GI/GII
14	PKV/CHGXQZ1/2021	2021	Guangxi, CHN	PV369273	PV369335	PV369397	GI/GII/GII
15	PKV/CHGXQZ2/2021	2021	Guangxi, CHN	PV369274	PV369336	PV369398	GI/GII/GII
16	PKV/CHGXLZ1/2023	2023	Guangxi, CHN	PV369275	PV369337	PV369399	GI/GII/GII
17	PKV/CHGXLZ2/2023	2023	Guangxi, CHN	PV369276	PV369338	PV369400	GI/GI/GII
18	PKV/CHGXLZ3/2023	2023	Guangxi, CHN	PV369277	PV369339	PV369401	GI/GI/GII
19	PKV/CHGXLZ4/2024	2024	Guangxi, CHN	PV369278	PV369340	PV369402	GI/GI/GI
20	PKV/CHGXLZ5/2024	2024	Guangxi, CHN	PV369279	PV369341	PV369403	GII/GI/GII
21	PKV/CHGXLZ6/2024	2024	Guangxi, CHN	PV369280	PV369342	PV369404	GII/GI/GI
22	PKV/CHGXLZ7/2024	2024	Guangxi, CHN	PV369281	PV369343	PV369405	GII/GII/GII
23	PKV/CHGXLZ8/2024	2024	Guangxi, CHN	PV369282	PV369344	PV369406	GII/GI/GII
24	PKV/CHGXLZ9/2024	2024	Guangxi, CHN	PV369283	PV369345	PV369407	GI/GI/GII
25	PKV/CHGXGL1/2022	2022	Guangxi, CHN	PV369284	PV369346	PV369408	GI/GI/GII
26	PKV/CHGXGL2/2022	2022	Guangxi, CHN	PV369285	PV369347	PV369409	GI/GI/GII
27	PKV/CHGXGL3/2025	2025	Guangxi, CHN	PV369286	PV369348	PV369410	GI/GI/GII
28	PKV/CHGXGL4/2025	2025	Guangxi, CHN	PV369287	PV369349	PV369411	GI/GI/GII
29	PKV/CHGXGG1/2021	2021	Guangxi, CHN	PV369288	PV369350	PV369412	GII/GI/GII
30	PKV/CHGXGG2/2021	2021	Guangxi, CHN	PV369289	PV369351	PV369413	GI/GI/GII
31	PKV/CHGXGG3/2023	2023	Guangxi, CHN	PV369290	PV369352	PV369414	GI/GI/GII
32	PKV/CHGXGG4/2023	2023	Guangxi, CHN	PV369291	PV369353	PV369415	GI/GII/GII
33	PKV/CHGXGG5/2024	2024	Guangxi, CHN	PV369292	PV369354	PV369416	GII/GI/GI
34	PKV/CHGXGG6/2024	2024	Guangxi, CHN	PV369293	PV369355	PV369417	GII/GI/GI
35	PKV/CHGXBH1/2025	2025	Guangxi, CHN	PV369294	PV369356	PV369418	GII/GII/GII
36	PKV/CHGXBH2/2025	2025	Guangxi, CHN	PV369295	PV369357	PV369419	GI/GII/GII
37	PKV/CHGXHZ1/2022	2022	Guangxi, CHN	PV369296	PV369358	PV369420	GII/GII/GI
38	PKV/CHGXHZ2/2022	2022	Guangxi, CHN	PV369297	PV369359	PV369421	GII/GII/GI
39	PKV/CHGXHZ3/2024	2024	Guangxi, CHN	PV369298	PV369360	PV369422	GI/GI/GII
40	PKV/CHGXBS1/2021	2021	Guangxi, CHN	PV369299	PV369361	PV369423	GII/GII/GII
41	PKV/CHGXBS2/2022	2022	Guangxi, CHN	PV369300	PV369362	PV369424	GII/GII/GII

42	PKV/CHGXBS3/2023	2023	Guangxi, CHN	PV369301	PV369363	PV369425	GII/GII/GI
43	PKV/CHGXBS4/2023	2023	Guangxi, CHN	PV369302	PV369364	PV369426	GI/GII/GII
44	PKV/CHGXBS5/2023	2023	Guangxi, CHN	PV369303	PV369365	PV369427	GI/GII/GII
45	PKV/CHGXBS6/2024	2024	Guangxi, CHN	PV369304	PV369366	PV369428	GI/GI/GI
46	PKV/CHGXWZ1/2023	2023	Guangxi, CHN	PV369305	PV369367	PV369429	GII/GII/GI
47	PKV/CHGXWZ2/2023	2023	Guangxi, CHN	PV369306	PV369368	PV369430	GII/GII/GI
48	PKV/CHGXLB1/2022	2022	Guangxi, CHN	PV369307	PV369369	PV369431	GII/GII/GII
49	PKV/CHGXLB2/2024	2024	Guangxi, CHN	PV369308	PV369370	PV369432	GII/GII/GII
50	PKV/CHGXFCG1/2024	2024	Guangxi, CHN	PV369309	PV369371	PV369433	GI/GI/GII
51	PKV/CHGXFCG2/2024	2024	Guangxi, CHN	PV369310	PV369372	PV369434	GI/GI/GII
52	PKV/CHGXFCG3/2024	2024	Guangxi, CHN	PV369311	PV369373	PV369435	GI/GII/GII
53	PKV/CHGXCZ1/2022	2022	Guangxi, CHN	PV369312	PV369374	PV369436	GI/GII/GII
54	PKV/CHGXCZ2/2022	2022	Guangxi, CHN	PV369313	PV369375	PV369437	GI/GII/GII
55	PKV/CHGXCZ3/2024	2024	Guangxi, CHN	PV369314	PV369376	PV369438	GI/GII/GII
56	PKV/CHGXCZ4/2024	2024	Guangxi, CHN	PV369315	PV369377	PV369439	GII/GII/GII
57	PKV/CHGXCZ5/2024	2024	Guangxi, CHN	PV369316	PV369378	PV369440	GII/GI/GII
58	PKV/CHGXCZ6/2024	2024	Guangxi, CHN	PV369317	PV369379	PV369441	GI/GII/GII
59	PKV/CHGXCZ7/2024	2024	Guangxi, CHN	PV369318	PV369380	PV369442	GI/GII/GII
60	PKV/CHGXHC1/2021	2021	Guangxi, CHN	PV369319	PV369381	PV369443	GII/GII/GII
61	PKV/CHGXHC2/2021	2021	Guangxi, CHN	PV369320	PV369382	PV369444	GII/GII/GII
62	PKV/CHGXHC3/2023	2023	Guangxi, CHN	PV369321	PV369383	PV369445	GI/GII/GII

Table S3. The information on the PKV reference strains used for Maximum Clade Credibility (MCC) tree.

No.	Virus strain	Date	Origin	Accession Number	Group
1	AH-42	2022	Anhui, CHN	OM274026	GI
2	Y-1-CHI	2010	Beijing, CHN	GU292559	GII
3	CH/HNXX-4/2012	2012	Gansu, CHN	JX401523	GI
4	swKoV CH441	2012	Gansu, CHN	KF539763	GI
5	JXJC2015	2015	Guangdong, CHN	KY234500	GII
6	Guangxi/G9/2012/CH	2012	Guangxi, CHN	MF062447	GII
7	Guangxi/G11/2012/CH	2012	Guangxi, CHN	MF062446	GI
8	Guangxi/G13/2013/CH	2013	Guangxi, CHN	MF062444	GI
9	Guangxi/G12/2012/CH	2012	Guangxi, CHN	MF062445	GII
10	swine/JX-1/2013/China	2013	Heilongjiang, CHN	MT125682	GI
11	CH/DX/2012	2012	Heilongjiang, CHN	KJ452348	GI
12	XX	2012	Henan, CHN	KC204684	GI
13	WUH1	2011	Hubei, CHN	JQ692069	GII
14	Wuhan2020	2020	Hubei, CHN	OK315318	GII
15	JS-02a-CHN/2014/China	2014	Jiangsu, CHN	KP260507	GI
16	ch-kobu/2008/China	2008	Jiangsu, CHN	KF695124	GI
17	Chgz2	2015	Jiangsu, CHN	MG800804	GII
18	CH/KB-1/2014	2014	Jiangxi, CHN	KM051987	GI
19	JXAT2015	2015	Jiangxi, CHN	KY234499	GI
20	K-11/2012/CH	2012	Jilin, CHN	KC414936	GII
21	SH-W-CHN/2010/China	2010	Shanghai, CHN	JN630514	GI
22	BSF2	2021	ZAF	OM105002	GII
23	XJ1904-34-PKV	2022	Xinjiang, CHN	ON007233	GI
24	SP-VC18	2017	ESP	ON792976	GII
25	SP-VC4	2017	ESP	ON792975	GII
26	KobuV/Pig-wt/ESP/C429/2017	2017	ESP	MK962326	GII
27	KobuV/Pig-wt/ESP/VT25C/2017	2017	ESP	MK962325	GII
28	FoPro-213.14/GER/2018	2018	GER	MZ334483	GII
29	PKV_GER_L00919-K17_14-02_2014	2014	GER	LT898428	GII
30	06-2017-Medj	2017	HR	OQ595081	GII
31	K-30-HUN/2008/HUN	2008	HUN	GQ249161	GII
32	SZ1M-F/PKV/HUN/2013	2013	HUN	MN807751	GII
33	WB-1-HUN/2011/HUN	2011	HUN	JX177612	GII
34	PoKoV/Ishi-Im6/JPN/2015	2015	JPN	LC210615	GI
35	PoKoV/Ishi-Im5/JPN/2015	2015	JPN	LC210614	GI
36	PoKoV/Bu9-10/JPN/2014	2014	JPN	LC210611	GII
37	PoKoV/Bu7-9/JPN/2014	2014	JPN	LC210610	GII
38	EdoMex/2018/205	2018	MEX	MT211964	GI
39	OH/RV50/2011	2011	USA	KM977675	GI
40	OH/RV11/2011	2011	USA	MF506730	GII
